# The case of triethylammonium cation loss during purification of certain nucleotide analogues: a cautionary note

**DOI:** 10.1007/s00216-014-8397-0

**Published:** 2014-12-27

**Authors:** Krystian Kolodziej, Joanna Romanowska, Jacek Stawinski, Adam Kraszewski, Michal Sobkowski

**Affiliations:** Institute of Bioorganic Chemistry, Polish Academy of Sciences, Noskowskiego 12/14, 61-704 Poznań, Poland

**Keywords:** Cation loss, Anion to cation ratio, Triethylammonium cation, Phosphates, Nucleotide analogues, Purification techniques, Solvent evaporation

## Abstract

Nucleotides, their analogues, and other phosphate esters and phosphoramidates often contain the triethylammonium cation as a counterion. We found that this may be lost during chromatographic purification or concentration of solutions, yielding products in acidic forms or containing sub-stoichiometric amounts of the counterion. This in turn may be detrimental, e.g., due to possible decomposition of a compound or inaccurate sample preparation. Correlations between the structure of studied compounds and their susceptibility for cation loss were analyzed. Modifications in preparative techniques were developed to obtain the studied compounds with stoichiometric anion to cation ratios.

ᅟ

**Electronic supplementary material:**

The online version of this article (doi:10.1007/s00216-014-8397-0) contains supplementary material, which is available to authorized users.

**Graphical Abstract:**
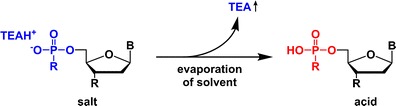
Triethylammonium salts of phosphate esters and phosphoramidates may lose the cationic component during chromatography or evaporation of solvent

## Introduction

Ionizable phosphorus esters of biomolecules, e.g., nucleotides and their analogues, are often isolated in the form of salts with the triethylammonium cation (TEAH^+^). A key feature of such salts is their significantly better solubility in organic solvents in comparison to free acids or metal salts, which is of particular importance for reactions requiring anhydrous conditions. For example, the sodium salt of 5′-*O*-dimethoxytritylthymidine *H-*phosphonate dissolves in dichloromethane marginally, while for its triethylammonium salt, concentrations of >1 M can be achieved readily.

In a laboratory practice, the presence of an excess of TEAH^+^ cation in purified organic salts (e.g., nucleotide derivatives) may be occasionally noted, typically due to a TEA·HCl impurity or incomplete removal of triethylamine (TEA). Recently, however, we encountered the opposite phenomenon: during a purification procedure of pharmacologically active nucleoside (*N*-aryl)phosphoramidates, some of them were losing triethylammonium cation [1]. Such observation was not, to our knowledge, reported so far in the literature. It is worth realizing that the weight of the cation contributes significantly to the total molecular mass of a given compound (e.g., in a bis-TEAH^+^ salt of thymidine monophosphate, the cations account for almost 40 % of its mass), and its accidental non-stoichiometric amount in a product may compromise its accurate measurements. This is particularly important in chemical synthesis and manipulations of multi-milligram or more amounts of compounds, for which weighting is a typical method for quantification.^1^ Irrespective of the scale, changes in physical and chemical properties associated with conversion of salts into acids are possible. Here, we present the studies that we undertook on the phenomenon of cation loss.

## Results and discussion

**Fig. 1:**
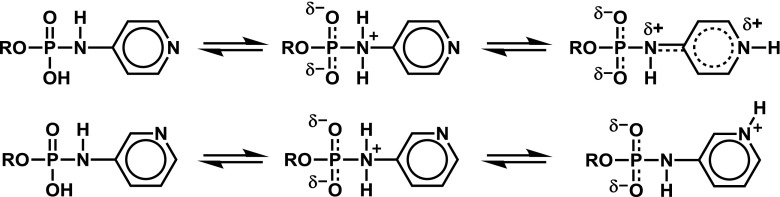
Possible protonation sites in *N*-(pyridin-4-yl) and *N*-(pyridin-2-yl) phosphoramidic acids. The rightmost tautomer of the *para* isomer is stabilized additionally by resonance, which is not possible for the *meta* isomer

**Fig. 2:**
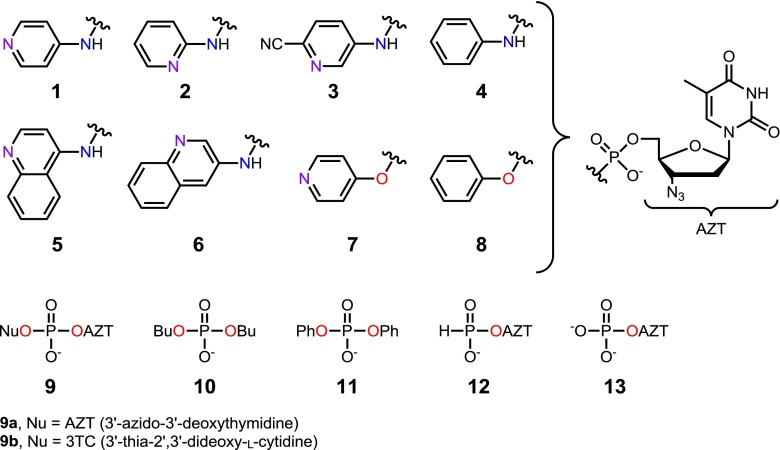
Structures of phosphoesters and amides studied in this work

Our previous observations of sub-stoichiometric amounts of TEAH^+^ cation present in purified samples concerned (*N*-aryl)phosphoramidates. For example, azidothymidine [*N*-(pyridin-4-yl)]phosphoramidate **1** (4AP-AZT, Fig. 2) and its *ortho* isomer were found to be particularly prone to losing the cation, and this was assigned to stabilization of the free acid due to charge delocalization in one of the tautomeric forms possible (Fig. 1) [1].

However, decomposition of salts which results in separation of their acidic and basic parts may occur also during their chromatographic isolation. Both processes require dissociation of the salt into neutral components, acid and base (Eq. 1), and the subsequent removal of free amine, either due to its different mobility on silica-gel or due its volatility during evaporation. The resulting decrease in concentration of the amine shifts the equilibrium (1) to the right.

1 $$ {\mathrm{A}}^{-}{\mathrm{R}}_3{\mathrm{NH}}^{+}\rightleftharpoons \mathrm{A}\mathrm{H} + {\mathrm{R}}_3\mathrm{N} $$

Both processes were investigated for several phosphates and phosphoramidates (Fig. 2). A special care was taken to ensure precise 1:1 ratio of the cation and the anion in the studied compounds. Thus, new purification procedures were developed to remove a potential TEAH^+^ excess (e.g., such as TEA·HCl, which is a by-product of the condensation step) and to prevent an accidental loss of the phosphate TEAH^+^ counterion. In the case of esters **8**–**12**, standard chromatographic purification [2] was followed by a Dowex (H^+^ form) cation exchange column which converted any chlorides into HCl, removed simply during the subsequent concentration. The desired esters in acidic forms were neutralized with a small excess of TEA and lyophilized to afford the corresponding TEAH^+^ salts. In the case of phosphoramidates **1**–**6**, this protocol could not be used due to their partial decomposition in the presence of the strongly acidic Dowex resin. However, the TEA·HCl contamination could be removed by silica-gel chromatography using ethyl acetate-TEA-MeOH elution system.^2^ The obtained amides were lyophilized from aqueous solution containing a small excess of TEA. Apparently, during freeze drying, the equilibration (1) is not operating and the TEAH^+^ cation was found in the final products in stoichiometric ratios. For experimental details, see the Electronic Supplementary Material (ESM).

The putative chromatographic separation of acidic and basic components of organic salts was studied for TEAH^+^ salts of phosphoramidate **1**, 3′-azidothymidine [*N*-(quinolin-3-yl)]phosphoramidate **6** (3AQ-AZT), di(3′-azidothymidine) phosphate **9a**, and diphenyl phosphate **11** (DPP), using toluene-MeOH and DCM-MeOH eluents (for structures, see Fig. 2).

In order to preclude evacuation of TEA during evaporation of the solvents, the amine was trapped in a form of TEA·HCl by acidification of the collected fractions with a slight excess of HCl before concentrated.^3^

**Fig. 3:**
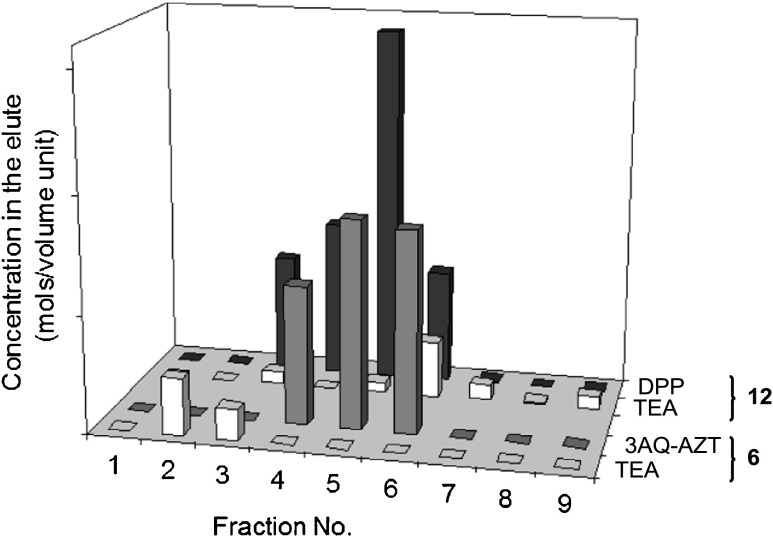
Elution profiles of TEAH^+^ salts of 3AQ-AZT **6** and DPP **11** during silica-gel chromatography with 1:1 (*v*/*v*) toluene-MeOH system. The *bars* reflect relative molar concentrations determined according to integration of the appropriate ^1^H NMR signals. No attempts were made to establish absolute concentrations

The results varied only slightly from case to case, and some general conclusions could be drawn: (i) the TEAH^+^ cation exclusion was not limited to heteroaromatic phosphoramidates, but it was observed for all the salts investigated; (ii) the anion to cation ratio was different in individual fractions collected, suggesting that these components migrated, at least partly, independently; and (iii) most (or all in some cases) of TEA was not recovered and was presumably bound to silica-gel (Fig. 3). The assumption of partial or total capture of the basic component by silica-gel was in agreement with further observations that the quantity of the TEA eluted varied accordingly to the sample to silica-gel ratio as well as to the amount of TEA added to the solution of a sample in simulations of crude reaction mixtures.

The second assumed possibility of exclusion of the TEAH^+^ cation from the salts studied is co-evaporation of the corresponding amine with solvents during concentration. This process was supposed previously to occur for pyridyl phosphoramidates **1** [1]. To verify these speculations and to evaluate generality of this phenomenon, a range of various types of phosphate esters and amides were subjected to evaporation with added solvents. To this end, samples of tested compounds were dissolved in 1:1 (*v*/*v*) toluene-methanol and evaporated to dryness in a rotary evaporator. The procedure was repeated up to nine times. The phosphate to TEAH^+^ ratio was estimated by comparing integration values of ^1^H NMR signals of ribose (or alkyl/aryl for **10** and **11**) and TEAH^+^ protons.

**Fig. 4:**
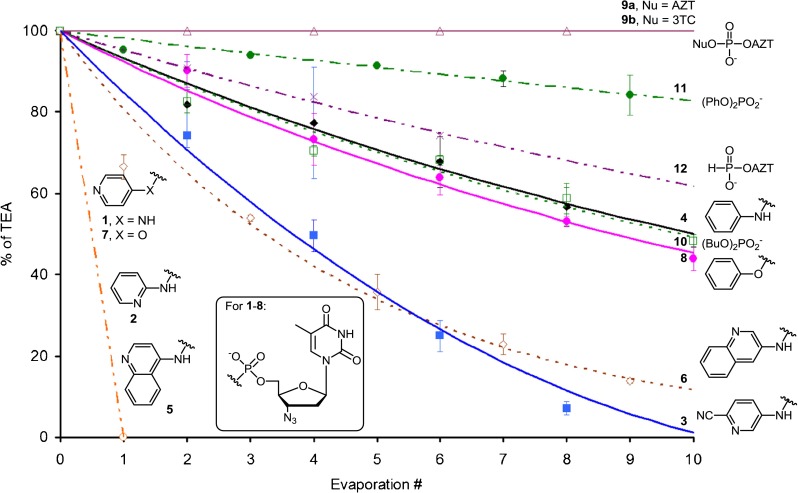
The loss of TEAH^+^ cation during repeated evaporation of solutions of phosphoesters and amides in 1:1 (*v*/*v*) toluene-MeOH system. The degree of cation loss during evaporation of a solvent depends strongly on the experimental conditions (e.g., bath temperature, vacuum, resistance of flow of the vapors, flask size, shape, and filling volume). While under strict experimental regime it was possible to gain a reasonable reproducibility, the results given here should be treated qualitatively (rather than quantitatively) as a general propensity of a given system to lose the cationic component

The results of evaporation experiments are collected in Fig. 4 (see also Figs. S1 to S3 in the ESM). Apart from dinucleoside phosphates, all the studied compounds were losing to some extent their TEAH^+^ cations, including nucleoside aryl ester **8**, dibutyl ester **10**, diphenyl ester **11**, and AZT *H-*phosphonate **12**, as well as all phosphoramidates.

Our initial expectations to find a clear-cut correlation between acidity of the P-O-H function and propensity of a derived salt to lose the cation were fulfilled only partly. The p*K*_a_ values for diesters of phosphoric acid (**8**–**11**) should fall in the range of 1–2 (due to lack of literature data, only estimation of p*K*_a_’s according to reported data [3–5] for the related species could be done), while that of amide esters (as in **4**), 3–4 [6]. However, phosphoramidate **4** did not lose the cation more rapidly than the apparently more acidic diesters **8** and **10**, and the spread of cation instability for esters **8**–**12** was rather large despite their expected similar acidity.

An additional basic nitrogen atom in a heteroaromatic ring (pyridyl and quinolinyl derivatives **1**–**3** and **5**–**6**) increased lability of the cation significantly, particularly for *ortho* and *para* derivatives, clearly indicating the importance of stabilization of the protonated species (cf. Fig. 1). Interestingly, the cation in both 4-aminopyridyl amide **1** and 4-hydroxypyridyl ester **7** was very labile. Since the aforementioned phenyl amide **4** and phenyl ester **8** also showed similar cation lability, it may be assumed tentatively that isostructural phosphate esters and phosphoramidates bind their cations with comparative strength.

While the TEAH^+^ cation appeared to be stable in dinucleotides of type **9** in the above experiments, for synthetic oligo(nucleoside phosphorothioates), a gradual loss of the TEAH^+^ cation has been observed during repeated evaporations (Sanghvi YS (Rasayan Inc.), personal communication). Admittedly, oligonucleotides are typically synthesized in sub-micromolar scale, and since their concentration is assessed usually by spectroscopic methods, the type of the counterion is rather insignificant. However, this is not the case for therapeutic antisense oligonucleotides, which are prepared routinely in multigram amounts. In that scale, their quantity is conveniently determined by weighting [7, 8], and proper accuracy of measurements requires that the stoichiometric contents of the cation are maintained.

**Fig. 5:**
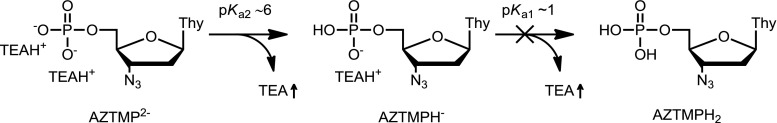
The loss of TEAH^+^ cation in bis-TEAH^+^ salt of AMP

In complementary experiments, we found that bis-TEAH^+^ salt of AZT monophosphate **13** lost one of the counterions during the first evaporation of the solvent, while the second one was fully resistant to multiple evaporations (Fig. 5). Such behavior was apparently governed by the estimated weak acidity of AZTMPH^−^ (p*K*_a_ ≈ 6) and rather strong of AZTMPH_2_ (p*K*_a_ ≈ 1) [9].

In several experiments with non-phosphate anions, we observed that TEAH^+^ salts of strong hydrochloric and *p*-benzenesulfonic acids were stable (evaporation of 20 mg/400 mL—no detectable cation loss), while that of benzoic acid (p*K*_a_ 4.2) was labile during evaporation of the same 1:1 (*v*/*v*) toluene-methanol solvent system (evaporation of 20 mg/400 mL—ca. 20 % cation loss). Thus, in these cases, the lability of the TEAH^+^ cation may be plausibly correlated with p*K*_a_ of the conjugated acids. The threshold value of p*K*_a_, below which the cation is stable, may be thus estimated as ca*.* 1. Since p*K*_a_’s of nucleotides are influenced by the structure of nucleoside [9], it may be speculated that p*K*_a_’s of dinucleoside phosphoric acids **9a** (H^+^ form) and **9b** (H^+^ form) are low enough to prevent the cation loss during evaporation, while those of mononucleoside species are above the threshold of the cation stability.

Additionally, it was found that the strongly basic and nonvolatile DBU (1,8-diazabicycloundec-7-ene) formed a fully stable salt with 4APy-AZT acid, while NH_4_^+^ or MeNH_3_^+^ cations were eliminated readily, as could be expected (evaporation of 20 mg/50 mL—complete cation loss). One should note that under mild experimental conditions used in our work (*t* ≤ 40 °C, *p* ≈ 1 Torr), we did not observe any mass loss of potentially labile TEA·HCl or NH_3_·HCl, although their cationic and anionic components are both volatile.

Finally, the influence of the solvents on the cation loss was tested for two phosphoramidates (TEAH^+^ salts), namely very labile **1** and moderately stable **6** (Table 1). A generally good correlation with the boiling point of the higher-boiling component is clear with an exception of water, which is apparently less effective in the cation removal.

**Table 1:** The percentage of initial stoichiometric contents of the TEAH^+^ cation after a single evaporation of various solvents and their mixtures (50 mL per 20 mg of a sample); standard deviation ca. 3–7

	DCE-MeOH 1:1 (*v*/*v*)	DCM-MeOH 1:1 (*v*/*v*)	H_2_O	PrOH-H_2_O 1:1 (*v*/*v*)	Dioxane-MeOH 1:1 (*v*/*v*)	Toluene-PrOH 1:1 (*v*/*v*)	Toluene-MeOH 1:1 (*v*/*v*)	DMF	Toluene-DMF 1:1 (*v*/*v*)
**1** (4AP-AZT)	70^a^	66	14	4	0	0	0	0	0
**6** (3AQ-AZT)	n.d.	100	100	100	100	92	90	37	39

Boiling points: DCM (dichloromethane), 40 °C; MeOH, 65 °C; DCE (1,2-dichloroethane), 84 °C; PrOH, 97 °C; water, 100 °C; 1,4-dioxane, 101 °C; toluene, 111 °C; DMF, 153 °C.

^a^Precipitation of **1** occurred after evaporation of ca. one half volume of the solvent mixture

Since the loss of cation during evaporation of solvents is assumed to result from evacuation of a volatile free base present in the solution due to the equilibrium (1), we anticipated that upon stopping the equilibration by freezing, it should be possible to isolate stoichiometric salts. To this end, the phosphoesters and amides obtained in acidic or partly acidic forms were dissolved in water containing ca. 20 % molar excess of TEA (as judged from the ^1^H NMR spectra) and freeze dried. We were delighted to see that after such treatment, the products had the expected cation to anion ratio.

Interestingly, incidents of non-stoichiometric amounts of the TEAH^+^ counterion in purified compounds can be found in the literature. For example, 5′-monophosphates of a series of nucleoside derivatives were obtained by high-performance liquid chromatography (HPLC) purification in triethylammonium bicarbonate buffer followed by a repeated lyophilization from water and were expected to be in the form of bis-TEAH^+^ salts [10]. According to the integration of the appropriate signals in ^1^H NMR spectra, in most cases, about two equiv. of the TEAH^+^ cation were present in the products, indeed. However, for two phosphate monoesters, only one equiv. of TEAH^+^ cation can be found in the spectra (triethylammonium salts of 3′-*O*-[*N*-For-Gly-l-β-amino-Ala-(β-carbamate)-l-Phe-OMe] and 3′-*O*-[*N*-For-l-Met-l-Lys-(ε-carbamate)-l-Ala-l-Ala-l-Phe-OMe] derivatives of 2′-deoxythymidine-5′-monophosphate) [10], and this suggests that during lyophilization, melting of the material could take place, allowing the equilibrium (1) to proceed. Conversely, four or more equiv. of TEA found in some other cases (triethylammonium salts of 3′-*O*-[*N*-For-l-Met-l-Lys-(ε-carbamate)-l-Lys-(ε-NH_2_)-OMe], 3′-*O*-[*N*-For-l-Met-l-Glu-(methyloxy-δ-carboxamide)-OMe], and 3′-*O*-[*N*-For-l-Met-l-Glu-(δ-ester)-l-Phe-OMe] derivatives of 2′-deoxythymidine-5′-monophosphate) [10] indicated incomplete removal of the base or its salts.

## Conclusions

During mild laboratory manipulations such as silica-gel chromatography or low-temperature concentration of solutions of nucleotide derivatives or in general, any salts containing protonated amines as counterions, the cations may be partly or totally eliminated and the products obtained may have sub-stoichiometric cation to anion ratio. This calls for attention for several reasons. First, the isolated acidic forms may have significantly different molecular masses than the expected salts, and this may compromise accuracy in weighting the samples, affecting actual concentration of prepared solutions or calculations of yields. Second, some physical properties of the compound may be affected, e.g., precipitation of less soluble acidic forms may occur during chromatography or concentration of solutions. Such unexpected solubility problems hampered severely the preparation of phosphoramidates of type **1** in our lab prior to identification of the phenomenon. Finally, decomposition in storage may take place if the products are isolated unintentionally in acidic forms, particularly in the case of phosphoramidates, which are prone to acid-catalyzed reactions, or for compounds containing other acid-labile residues, e.g., trityl-type protecting groups. Importantly, partial loss of the cation may be overlooked easily since typically it does not give rise to any additional signals in NMR spectra or peaks in HPLC chromatograms, and only careful quantification of the areas of ^1^H NMR signals may reveal sub-stoichiometric amounts of cations.

The risk of cation loss may be diminished by avoiding evaporation of large quantities of high-boiling solvents. The alternatives include choosing more volatile solvents or other techniques of isolation of products, e.g., freeze drying, precipitation, or crystallization, although these are not always applicable. The TEAH^+^ cation can be re-introduced quantitatively by lyophilization from TEA-enriched solutions.

## Electronic supplementary material

Below is the link to the electronic supplementary material.

ESM 1 (PDF 221 kb)

## Footnotes

1 On the contrary, the methods used in biochemical experiments for determination of the amount of a sample, e.g., those based on enzyme activity or optical density units, are generally not affected significantly by the presence or lack of counterions.

2 Among a variety of solvents examined, the MeOH-EtOAc-TEA solvent system was the only one in which TEA·HCl could be separated from the desired compounds.

3 TEA·HCl was found to be not volatile under the conditions used.
