# Supplementary material for: The case of triethylammonium cation loss during purification of certain nucleotide analogues: a cautionary note
Source: Anal Bioanal Chem. 2014 Dec 27;407(6):1775–80. doi: 10.1007/s00216-014-8397-0 (PMC4329187; doi:10.1007/s00216-014-8397-0)
Supplement: Supplementary file 1 — (PDF 221 kb) [file 216_2014_8397_MOESM1_ESM.pdf]

Analytical and Bioanalytical Chemistry

Electronic Supplementary Material

The case of triethylammonium cation loss during purification of certain nucleotide analogues: a cautionary note

Krystian Kolodziej, Joanna Romanowska, Jacek Stawinski, Adam Kraszewski,
Michal Sobkowski

Materials and methods

All reagents and solvents were of analytical grade, obtained from commercial suppliers and used without further purification. NMR spectra were recorded on Varian Unity BB VT 300 MHz or Bruker Avance II 400 MHz machines. Preparative column chromatography was monitored by TLC using Merck silica gel 60 F_{254} plates and UV light at 254 nm. Nucleotide analogues **1-9** and **12** were prepared according to the published procedures,[1;2] however, the purification procedures were modified (*vide infra*). The anion:cation ratios were calculated from integration of the appropriate signals in ^1H NMR spectra, apart from TEA·HCl and NH_4·HCl, for which gravimetric analysis was used.

*Purification of phosphoramidates **1-6***

Phosphoramidates **1-6** were purified by silica-gel chromatography using 0-20% gradient of MeOH in ethyl acetate containing 5% TEA. TEA·HCl was eluted prior to the desired products and was observed as a faint spot on TLC plates under 254 nm UV light. Fractions containing the pure product were pooled and evaporated to dryness under reduced pressure. The residue was dissolved in a minimal amount of water containing ca. 2 equiv. (in respect to phosphoramidate) of TEA and lyophilized. Stoichiometric amount of the $TEAH^+$ cation (1 equiv.) was confirmed by ^1H NMR spectroscopy.

*Purification of phosphates **7-9** and H-phosphonate **12***

Esters **7-9** and **12** purified as described previously[1] were dissolved in water and passed through a Dowex (H^+ form) column. Fractions containing the pure product were pooled and evaporated to dryness under reduced pressure. The residue was dissolved in a minimal amount of water containing ca. 2 equiv. of TEA and lyophilized. Stoichiometric amount of the $TEAH^+$ cation (1 equiv.) was confirmed by ^1H NMR spectroscopy.

*Dibutyl phosphoric acid, $TEAH^+$ salt (**10**)*

Commercial dibutyl phosphoric acid was converted into $TEAH^+$ salt using Dowex ($TEAH^+$ form) column. The solvent was evaporated and the product dried under high vacuum overnight. Stoichiometric amount of the $TEAH^+$ cation (1 equiv.) was confirmed by ^1H NMR spectroscopy.

*Diphenyl phosphoric acid, $TEAH^+$ salt (**11**)*

Diphenyl phosphoric acid was obtained as white crystals by hydrolysis of diphenyl chlorophosphate in water, followed by evaporation, repeated evaporation with anhydrous

acetonitrile (three times) and drying under high vacuum. The free acid was converted into $TEAH^+$ salt using Dowex ($TEAH^+$ form) column. The solvent was evaporated and the product dried under high vacuum overnight. Stoichiometric amount of the $TEAH^+$ cation (1 equiv.) was confirmed by ^{1}H NMR spectroscopy.

*3'-azido-3'-deoxythymidine monophosphate (AZTMP), bis-TEAH⁺ salt (**13**)*

AZT monophosphate, mono-$TEAH^+$ salt was dissolved in a minimal amount of water containing ca. 4 equiv. of TEA (2 equiv. per each P–OH) and lyophilized. Stoichiometric amount of the $TEAH^+$ cation (2 equiv.) was confirmed by ^{1}H NMR spectroscopy.

Cation stability during chromatography

A 2 cm diameter column was filled with 50 g of silica-gel and equilibrated with 1:1 (v/v) toluene-MeOH solution. A 100 mg sample of a studied compound was dissolved in 1 mL of the same solution and applied to the column, which was washed subsequently with the same solvents in a isocratic mode. 50 mL fractions were collected, acidified with 100 μL of conc. HCl_{aq} (ca. 5 equiv. with respect to the total applied sample), evaporated to dryness, dissolved in 1 mL of d6-DMSO and analyzed by ^{1}H NMR spectroscopy.

Cation stability during evaporation

A 20 mg sample of a studied compound in 100 mL flask was dissolved in 50 mL of a given solvent(s) and evaporated to dryness (30 min) in a rotary evaporator connected to a membrane vacuum pump working at maximum vacuum, and using a 40 ºC heating bath. The residue was dissolved in D_2O, analyzed by ^{1}H NMR spectroscopy, returned to the evaporation flask, diluted by the working solvent and the evaporation procedure was repeated. The results are collected in Fig. 4 in the article. Exemplary ^{1}H NMR spectra recorded during evaporations are shown in Figs. S1 to S3.

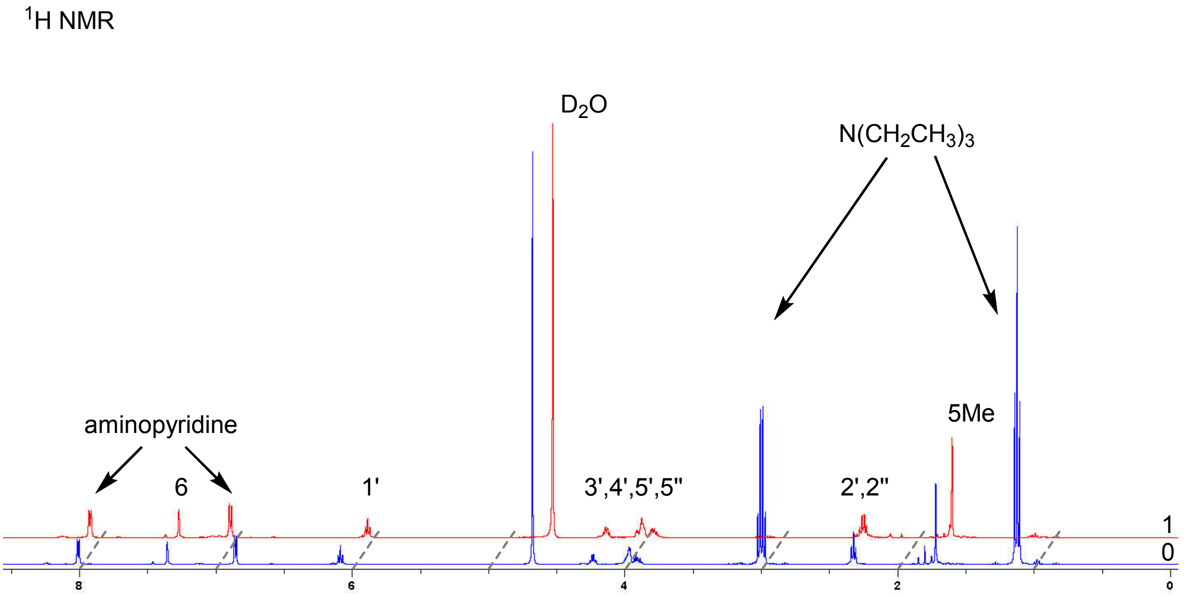

Fig. S1 ^{1}H NMR spectrum of 3'-azidothymidine [*N*-(pyridin-4-yl)]phosphoramidate, TEAH$^+$ salt **1** in D_2O overlapped with a spectrum after 1 evaporation of 1:1 (v:v) toluene-methanol solvent system

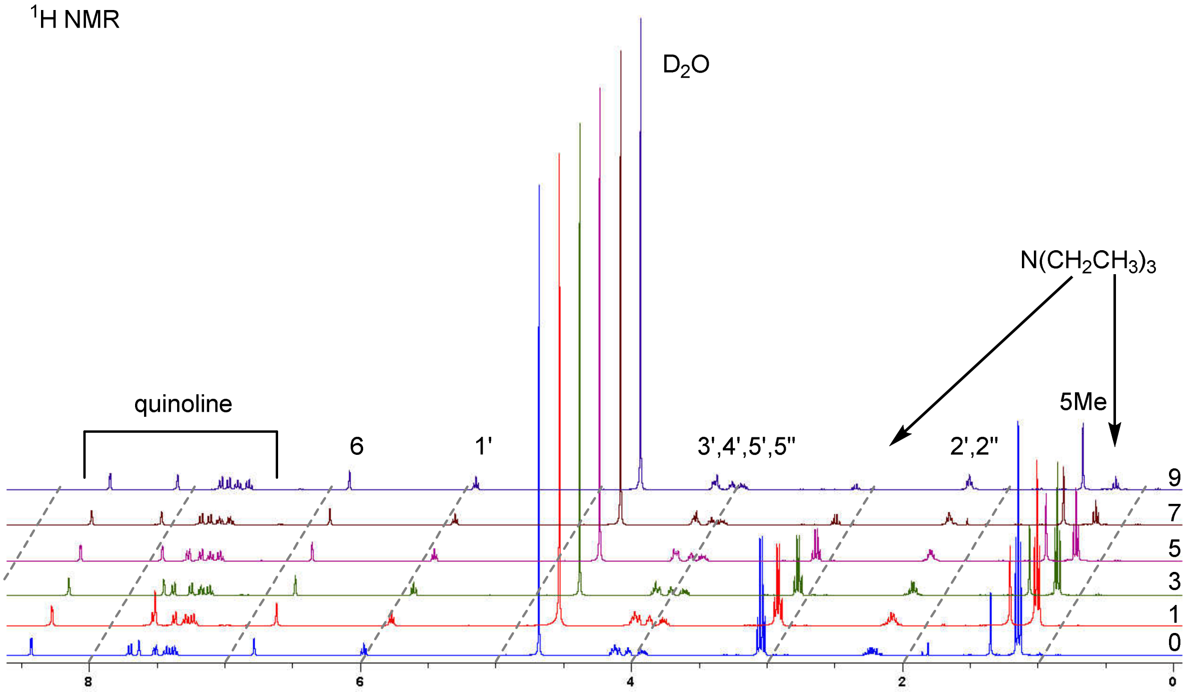

Fig. S2 ^{1}H NMR spectrum of 3'-azidothymidine [*N*-(quinolin-3-yl)]phosphoramidate, TEAH$^+$ salt **6** in D_2O overlapped with spectra after 1, 3, 5, 7, and 9 evaporations of 1:1 (v:v) toluene-methanol solvent system

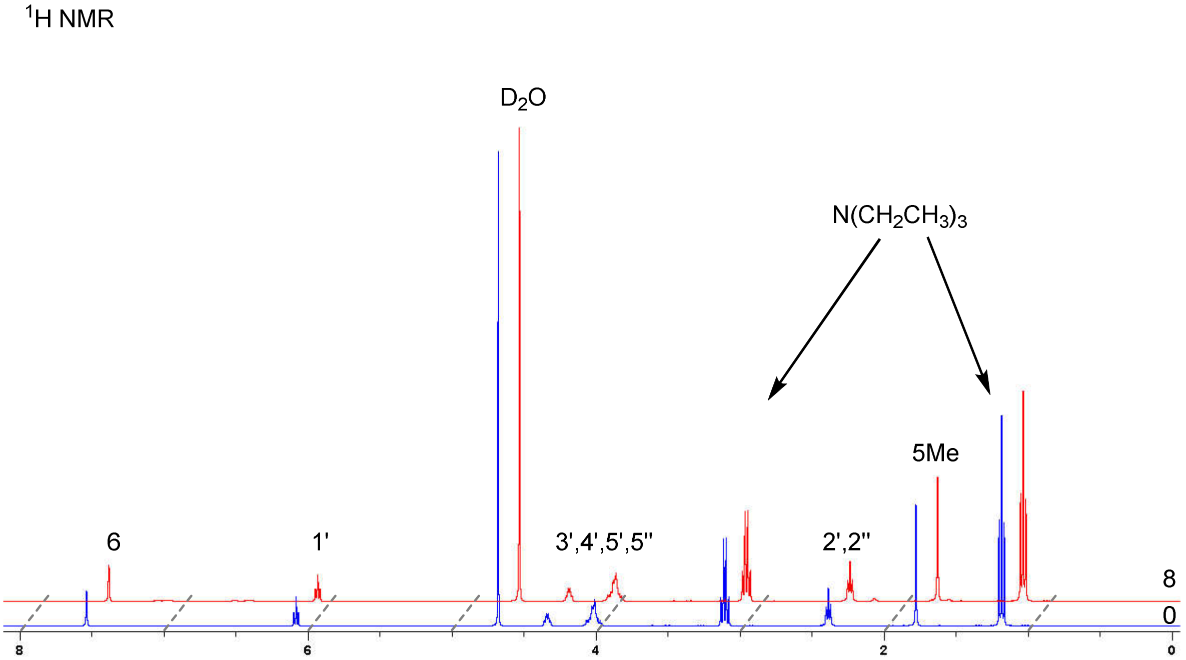

Fig. S3 ^1H NMR spectrum of bis(3'-azidothymidine) phosphate, $TEAH^+$ salt **9a** in D_2O overlapped with a spectrum after 8 evaporations of 1:1 (v:v) toluene-methanol solvent system

References

1. Romanowska J, Szymanska-Michalak A, Boryski J, Stawinski J, Kraszewski A, Loddo R, Sanna G, Collu G, Secci B, La Colla P (2009) Bioorg Med Chem 17:3489-3498

2. Romanowska J, Sobkowski M, Szymanska-Michalak A, Kolodziej K, Dabrowska A, Lipniacki A, Piasek A, Pietrusiewicz ZM, Figlerowicz M, Guranowski A, Boryski J, Stawinski J, Kraszewski A (2011) J Med Chem 54:6482-6491
